# Electron/energy co-transfer behavior and reducibility of Cu-chlorophyllin-bonded carbon-dots†

**DOI:** 10.1039/d0ra04958a

**Published:** 2020-08-26

**Authors:** Tian-Hao Ji, Xue-Li Li, Yongyun Mao, Zhipeng Mei, Yanqing Tian

**Affiliations:** Science College, Beijing Technology and Business University Beijing 100048 China jitianhao@th.btbu.edu.cn; Department of Materials Science and Engineering, Southern University of Science and Technology Shenzhen 518055 China tianyq@sustech.edu.cn

## Abstract

Cu-chlorophyllin-bonded carbon dots (CCPh-CDs) have been synthesized at room temperature, and the energy/electron co-transfer behavior between Cu-chlorophyllin molecules (CCPh) and carbon dots (CDs) is investigated *via* various techniques. The mean diameters of CDs and CCPh-CDs are 2.8 nm and 3.1 nm, respectively, measured by HRTEM. The absorption spectra of CCPh-CDs show two parts: the absorptions of CDs and CCPh are in the wavelength range of 300–500 nm. The PL spectra of CCPh-CDs exhibit very weak intensities, and with the decreasing of CCPh content on CDs, the corresponding intensity increases. Luminescent decay spectra show that the PL decay times of CCPh and CCPh-CDs with the highest CCPh content are single-exponentially fitted to be 3.20 ns and 12.64 ns, respectively. Furthermore, based on the electron transfer and reducibility of CCPh-CDs, Ag/Ag_2_O nanoparticles with a mean diameter of 10 nm can be easily prepared at room temperature under ultraviolet irradiation. The PL measurement result reveals that both electron transfer and FRET behavior take place from CCPh-CDs to Ag.

## Abstract 2

Cu-chlorophyllin-bonded carbon dots (CCPh-CDs) with/without Ag/Ag_2_O (CCPh-CD-Ag) were obtained and investigated by optical measurements and luminescence decay spectroscopy.

## Graphical abstract

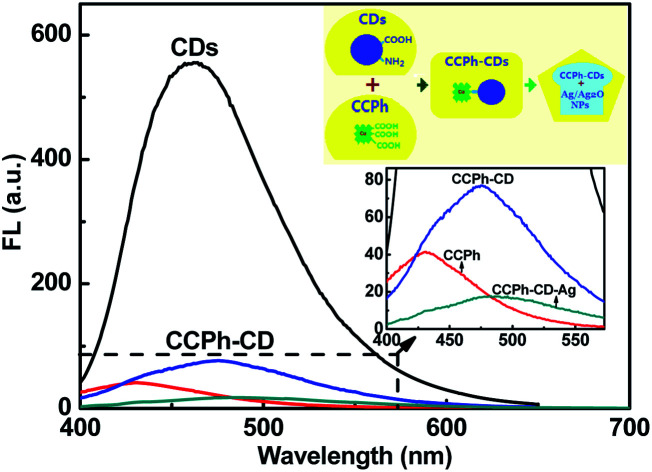

## Introduction

Over this decade, fluorescent carbon dots (CDs) have gained colossal attention and have undergone extensive research owing to their remarkable merits, including water solubility, high photo and chemical stability, low cost and toxicity, excellent biocompatibility, environmental friendliness, and strong photoluminescence.^1–5^ They show broad potential applications in many fields, including recognition sensors for various metal ions,^6–8^ bio-imaging in cells,^9,10^ photocurrent or photocatalytic materials,^11,12^ targeted drug delivery systems,^13,14^ photothermal therapies,^15,16^*etc.*

To achieve more remarkable performance, CDs are usually modified to couple with other materials, such as metal and nonmetal nanoparticles,^17–22^ as well as organic and polymeric materials.^23–30^ Shi *et al.* synthesized core–shell Au@CDs NPs with a shell CD-thickness of *ca.* 2 nm through the direct reduction of HAuCl_4_ by CDs, which enhanced surface Raman scattering measurements.^17^ Hu *et al.* combined zinc phthalocyaine (PcZn) with amine-functionalized CDs (N-CDs) in DMF by mechanical stirring and obtained nanocomposites, achieving photoinduced excited electron transfer from N-CDs to PcZn and high photocatalytic activity.^26^ Pal *et al.* synthesized CD-deposited polypyrrole-grafted chitosan (Ch-*g*-PPy/CDs), which exhibited much higher photocatalytic activity toward degradation of toxic 2-chloro phenol into small compounds in comparison with CDs, PPy/CDs and Ch-*g*-PPy.^30^ However, both PcZn/N-CDs and Ch-*g*-PPy/CDs could not be firmly bonded due to their weak interaction. Hence, their application is limited by the detachment of CDs from the corresponding nanocomposites in the solvent. In our previous work, the preparation and characterization of EDTA-bonded CDs have been investigated,^31^ and herein we investigate the synthesis and characterization of Cu-chlorophyllin-bonded carbon-dots (CCPh-CDs).

Chlorophylls have a tetrapyrrole framework with some peripheral side groups and chiral centers,^32^ thus they can be considered as a kind of special carbon dots. In general, natural chlorophylls are part-crystallographically organized in the protein matrix to realise light-harvesting antennas and reaction centers so that they can efficiently achieve intermolecular excitation energy transfer and electron transfer.^33^ Therefore, they were also employed as the photo-sensitizer or reducing agent for photodynamic therapy, solar cells and phototransistors. For example, Wang *et al.* reported chlorophyll molecules sensitized graphene-based phototransistors showing prominent photo-response;^34^ Manna *et al.* fabricated a nanohybrid of graphene oxide and chlorophyll molecules through π–π interaction, and confirmed that under white-light irradiation, such a nanohybrid accelerated the oxidation process of water;^35^ Asha *et al.* prepared binary oxide ZrO_2_–TiO_2_ films sensitized with natural chlorophyll;^36^ Zhang *et al.* recently used chlorophyll as the photo-sensitizer and prepared a composite with water-soluble graphene, showing an improved photodynamic therapy *in vitro*.^37^

In the work, we used Cu-chlorophyllin molecules with –COOH to synthesize CCPh-CDs through a reaction of –COOH with –NH_2_ on CDs at room temperature, and in addition, we prepared Ag/Ag_2_O nanoparticle-deposited CCPh-CDs in a mixture solution of Ag^+^ and CCPh-CDs under ultraviolet light irradiation. PL measurements show that the CCPh-CDs without or with Ag/Ag_2_O exist effective electron transfer and FRET dual-behaviors.

## Experimental section

### Reagents and instruments

All the chemicals are of analytical reagent grade and used without further purification. Citric acid monohydrate, ethylenediamine, dimethylsulfoxide (DMSO), copper chlorophyllin, 1-ethyl-3-(3-dimethylaminopropyl) carbodiimide hydrochloride (EDC), *N*-hydroxysulfosuccinimide sodium salt (S-NHS), ethanol and silver nitrate (AgNO_3_) were purchased from Sinopharm Chemical Reagent Co. Ltd.

The UV-Vis absorptions were measured with a Cary-60 UV-Vis spectrophotometer (Agilent, USA). The FL spectra of the samples in quartz cells were measured on a Cary Eclipse fluorescence spectrophotometer (Agilent, USA) using a Xe lamp as the excitation source in the same condition of measurements. The transmission electron microscope (TEM) images were captured on a Tecnai F30 instrument (FEI, USA). The X-ray powder diffraction (XRD) analysis was performed on an AXS-D2 diffractometer (Bruker, Germany) with Cu Kα radiation (*λ* = 1.5406 Å). The Cu^2+^ content in CCPh-CDs was measured by an Agilent-7900cx inductively coupled plasma mass spectrometer (ICP-MS). The decay lifetimes of the samples were measured at room temperature using an FLS920 lifetime spectrofluorophotometer.

### Preparation of Cu-chlorophyllin-bonded CDs

The precursor carbon dots (CDs) were prepared from citric acid and ethylenediamine in an autoclave, and the details can be found in the supporting information.†^31,38^ Subsequently, Cu-chlorophyllin-bonded carbon dots (denoted as CCPh-CDs) were synthesized as follows: (1) Cu-chlorophyllin (270.0 mg), S–NHS (800.0 mg) and EDC (350.0 mg) were dissolved in 60 mL DMSO at room temperature with stirring; (2) the CDs (500.0 mg) were added into the solution with stirring for 24 hours under light-proof condition; (3) the obtained solution was washed and centrifuged with ethanol for three times; (4) the solid product was vacuum-dried for 10 hours at 40 °C and the CCPh-CD1 (Cu^2+^ content: 1.21 wt%) has been synthesized.

For comparison, the other two samples with Cu^2+^ contents of 0.70 and 0.29 wt%, correspondingly marked as CCPh-CD2 and CCPh-CD3, were synthesized with the same procedure and condition of the CCPh-CD1 except for the added Cu-chlorophyllin amount (see Experiment section in ESI†).

### Preparation of CCPh-CD-Ag

**Fig. 1:**
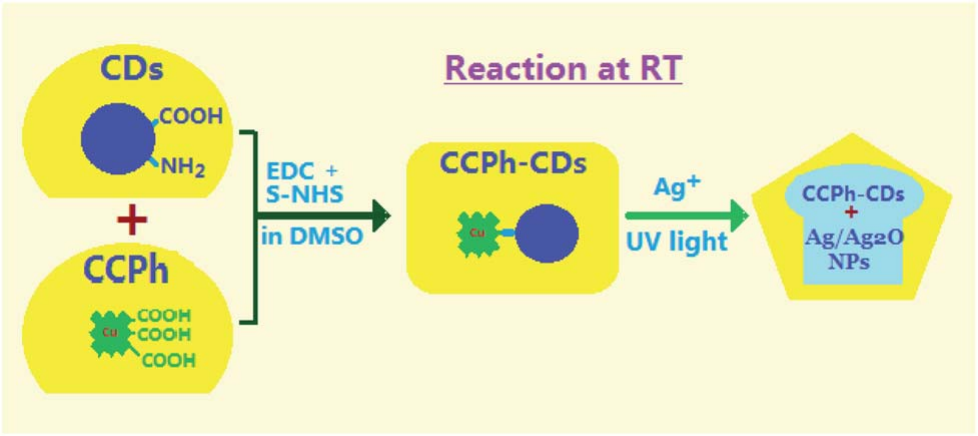
Schematic illustration of the preparation process of CCPh-CDs and CCPh-CD–Ag.

Based on CCPh-CD1, the CCPh-CD with Ag nanoparticles can be easily obtained as illustrated in Fig. 1. 200 mL DMF solution with 0.03 g of CCPh-CDs powder was first mixed in 100 mL aqueous solution with 0.085 g of AgNO_3_ at room temperature, and then the mixed solution was stirred for 30 min. Subsequently, the mixed solution was irradiated for 30 min under ultraviolet light with the wavelength of 360 nm. The final mixture was centrifuged and washed with water and ethanol for many times. Finally, the product (CCPh-CD–Ag) was vacuum-dried for 10 hours at 40 °C.

## Results and discussion

Cu-chlorophyllin-bonded carbon dots (CCPh-CDs) can be easily obtained *via* a room-temperature reaction under light-proof condition. The synthetic reaction was carried out in DMSO reagent with EDC and S–NHS for 24 hours so that Cu-chlorophyllin molecules can react adequately with C-dots.

**Fig. 2:**
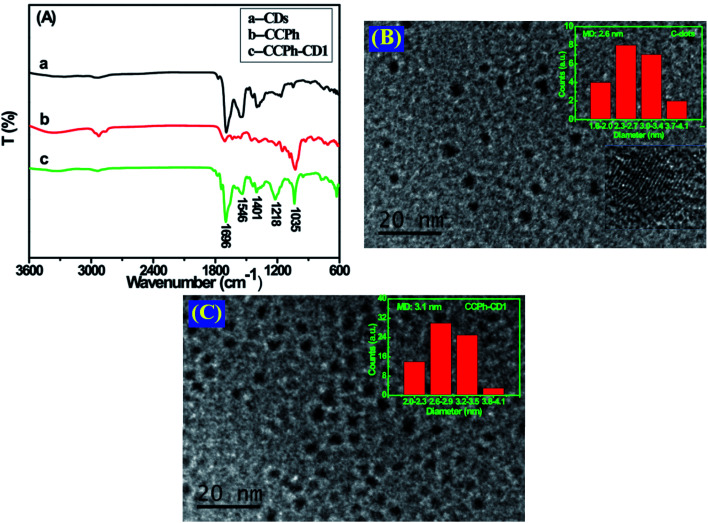
(A) FTIR spectra of CDs, CCPh and CCPh-CD1; (B) HRTEM image of CDs; (C) HRTEM image of CCPh-CD1. Insets are the corresponding diameter distributions.

In the FTIR spectra of the precursor CDs and CCPh-CD1 (Fig. 2A), the amide I and amide II bands appeared at around 1696 and 1546 cm^−1^, which indicated the vibration absorptions of C=O and N–H bands, confirmed the existence of the linker –NHCO– in these two samples.^39,40^ Since there are no N–H or –NHCO– in the CCPh structure, the peak at 1546 cm^−1^ was not observed. The observed CCPh-CD1 peaks at ∼ 1401 cm^−1^ and 1218 cm^−1^ is ascribed to C–H bending vibration and C–N stretching vibration, respectively. The absorption peak at around 1035 cm^−1^ is assigned to the C–O stretching vibration.^41^

The CDs and CCPh-CD1 nanoparticles were characterized using HRTEM and provided in Fig. 2B and C. The mean diameters of CDs and CCPh-CD1 are around 2.8 nm and 3.1 nm, respectively (provided in insets of Fig. 2B and C), indicating that the CCPh-CD1 particles are generally bigger than the CDs due to the bonding of CCPh on per C-dot. In addition, the crystal lattice of each C-dot can be observed clearly in the inset of Fig. 2B, confirming the integrality and orderliness of all atoms in C-dot.

**Fig. 3:**
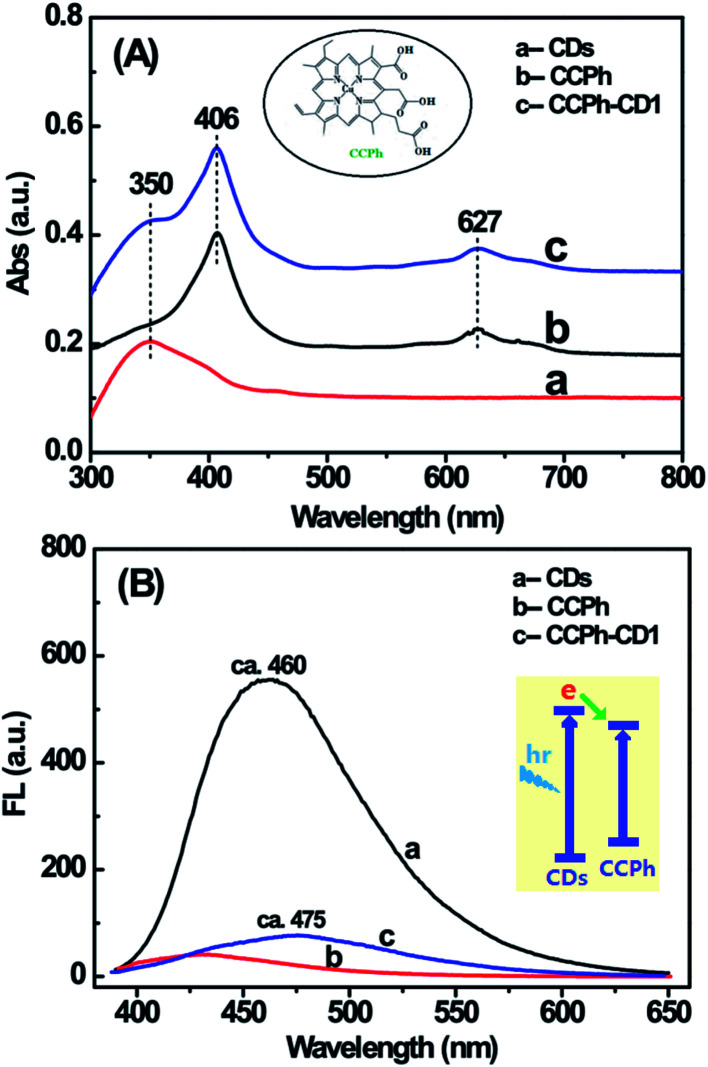
UV-Vis absorption (A) and PL spectra (B) of CDs, CCPh and CCPh-CD1 under excitation at 380 nm. Insets in (A) and (B) are CCPh molecular structure and FRET diagram between C-dot and CCPh, respectively.

Two UV-Vis absorption features of the CCPh-CD1 are observed (shown in Fig. 3A): the adsorption of CDs and CCPh. For CCPh-CD1, the peak at around 350 nm is assigned to the absorption of CDs; the peaks at around 406 and 627 nm are ascribed to the absorptions of S-band and Q-band of the porphyrin ring, respectively.^42^ In addition, their dilute solutions were measured for FL properties right after the UV-Vis absorption measurements. By comparison, we can qualitatively explain the PL intensity based on the similar intensities of the three highest absorption peaks. Under the excitation at 380 nm, the PL spectra of the three samples (Fig. 3B) differed obviously in the range of 400–600 nm, in which the CDs exhibited the strongest PL. After CCPh molecules were bonded to CDs and formed the CCPh-CD1, the PL intensity remarkably decreased owing to both the Förster resonance energy transfer (FRET) and the electron transfer behaviors between CCPh and C-dots. It is for such transfer behaviors from C-dots to CCPh that the CCPh-CD1 shows very weak PL intensity.

**Fig. 4:**
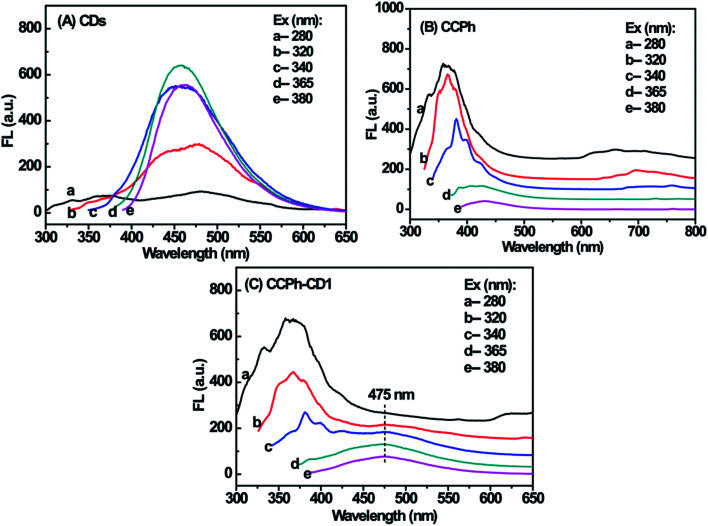
PL spectra of CDs (A), CCPh (B) and CCPh-CD1 (C) upon five different excitation wavelength.

The PL behaviors of CDs, CCPh and CCPh-CD1 at different excitation wavelength are presented in Fig. 4. The strongest fluorescent peaks among the CDs curves are those with excitation wavelength of 340, 365 and 380 nm, which exhibits similar intensities (Fig. 4A), and whereas they are significantly different from those of the CCPh and CCPh-CD1. The spectra of the CCPh-CD1 was generally similar to those of CCPh except for the widely weak PL peak at ∼475 nm (Fig. 4B, C and S-Fig. 1†). Such results show that under ultraviolet irradiation, C-dots have minor affection on the optical properties of the CCPh-CD1, which is due to the higher CCPh content.

**Fig. 5:**
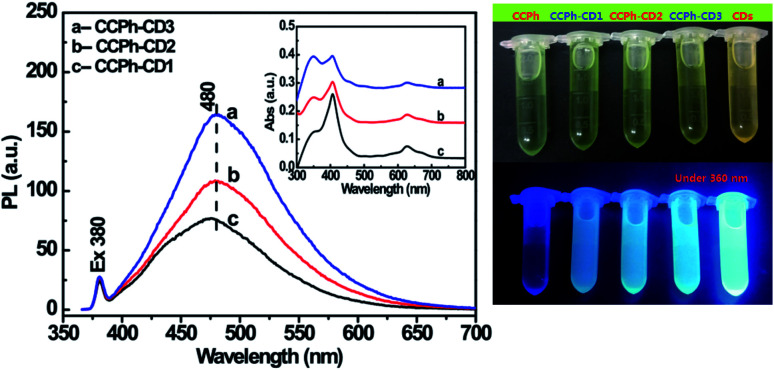
(Left) FL spectra of the three samples CCPh-CD1, CCPh-CD2 and CCPh-CD3 under 380 nm of excitation wavelength. Inset: the corresponding absorption spectra. (Right) Two pictures of the five samples CCPh, CCPh-CD1, CCPh-CD2, CCPh-CD3 and CDs irradiated under sunlight (top) and ultraviolet light of 360 nm (bottom).

To further investigate the electron transfer and FRET phenomena from CDs to CCPh, CCPh-CD2 with 0.70 wt% Cu^2+^ and CCPh-CD3 with 0.29 wt% Cu^2+^ were synthesized for comparison. Their PL spectra are provided in Fig. 5. Compared to the CCPh-CD1 (1.21 wt% Cu^2+^) under the concentration estimated from their absorptions in the inset, with the decrease of Cu^2+^, PL intensities at *ca.* 480 nm gradually increase under the excitation wavelength of 380 nm. It means that the increase of CCPh molecules bonded with per C-dot causes the decrease of the PL intensity, indicating that CCPh molecules lead to lower PL intensity. Thus, with the increase of bonded CCPh, the more electrons and electron transfer may take place from CDs to CCPh. Such results also show that under the irradiation of near ultraviolet light, more CCPh molecules on CDs can increase the absorption of S-band of the porphyrin ring.

**Fig. 6:**
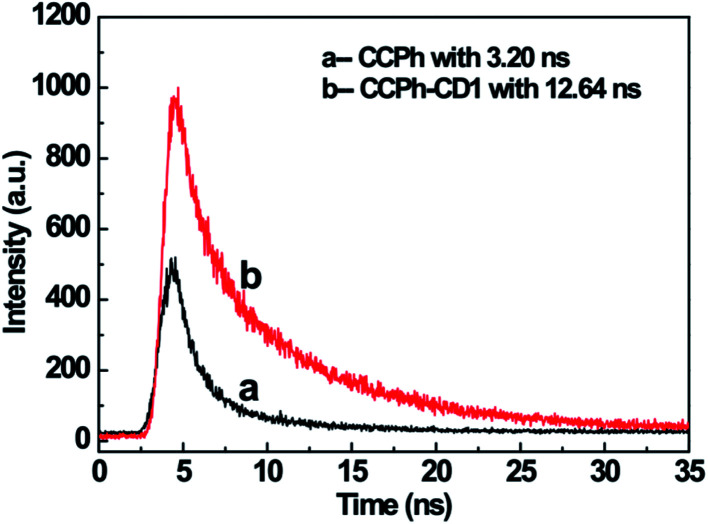
Luminescence decay spectra of CCPh (a) and CCPh-CD1 (b) with the excitation wavelength of 360 nm.

The luminescent decay spectra of CCPh and CCPh-CD1 with the excitation at 360 nm revealed the transfer properties of the photo-induced electron (Fig. 6). The PL decay (exciton lifetime) time of the CCPh and CCPh-CD1 were exactly single-exponentially fitted to be 3.20 ns and 12.64 ns, respectively. The exciton lifetime of the CCPh is similar to that of the C-dot precursor, reported as 3.6 ns,^43^ which is assigned to the radiative recombination of electron transfer within one C-dot. Whereas, the longer exciton lifetime of the CCPh-CD1 should be resulted from the electron transfer between the linked CCPh and C-dot. As the bandgap of the C-dot is wider than that of the CCPh, the excitation electron in C-dot will transfer to the linked CCPh, causing the decrease of the PL intensity of the C-dot. Such a deduction is in good agreement with the experiment result in Fig. 5. It is well known that C-dots exhibit weak oxidation or reduction because they can act as both excellent electron acceptors and electron donors.^31,44–47^ Recently, using C-dots as a catalytic reductant and capping agent, Wang *et al.* have successfully prepared stable silver nanoparticles (Ag NPs).†^46^ Since the plasmon absorption of Ag NPs appears mainly in the wavelength range of 350–500 nm, there exists not only electron transfer but also FRET behavior between C-dots and Ag NPs.^48^ Herein, in order to extend the application of C-dots with CCPh, we have also tried to reduce Ag^+^ to form Ag NPs in DMF solution of CCPh-CD1 under 360 nm irradiation for about half-hour. PL measurement results confirmed that the ultraviolet irradiation under such an experimental condition would not break the structure of the CDs, CCPh and CCPh-CD (S-Fig. 4 and 5†).

**Fig. 7:**
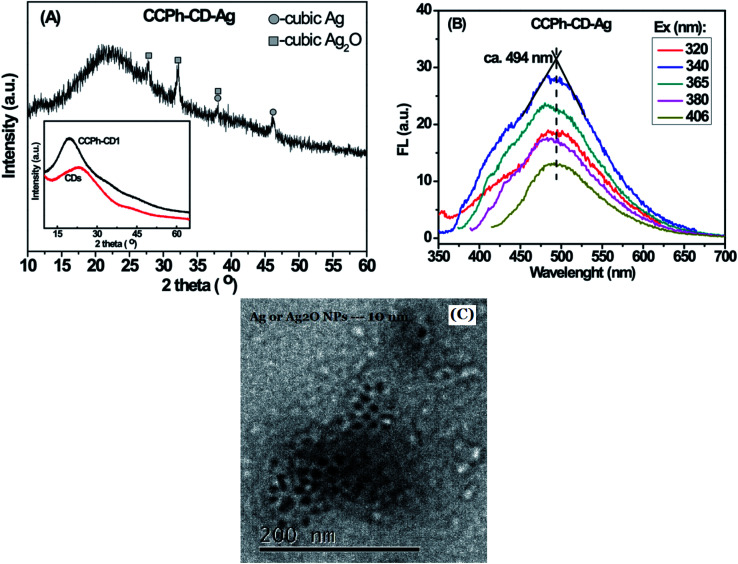
Characterization of CCPh-CD-Ag: (A) XRD pattern; (B) PL spectra under different excitation wavelength; (C) TEM image. Inset is the XRD patterns of CDs and CCPh-CD1.

The CCPh-CD-Ag nanocomposite was measured using XRD, PL and TEM as shown in Fig. 7. XRD pattern of CCPh-CD-Ag reveals the existence of cubic Ag and cubic Ag_2_O phases, indicating that the CCPh-CD1 under near ultraviolet irradiation can effectively reduce Ag^+^ to Ag. There has not been reasonably explained how Ag_2_O is formed, however, it could be related to hydroxide free radical OH˙ resulted from ultraviolet light.^49^ Owing to the Ag and Ag_2_O, the PL peaks of the CCPh-CD–Ag exhibit relatively weaker intensities in comparison with that of the CCPh-CD1 in Fig. 4C and S-Fig. 1,† which showing both electron transfer and FRET from CCPh-CD1 to Ag. Meanwhile, a slight red-shift of the peaks from 475 nm for CCPh-CD1 to 494 nm for CCPh-CD–Ag are observed, indicating the interaction between CCPh-CD1 and Ag NPs. In addition, the two similar FTIR spectra of the CCPh-CD1 and CCPh-CD1 in S-Fig. 6† reveal that ultraviolet irradiation and Ag-NPs formation barely affect the structure of CCPh-CD. TEM image of the CCPh-CD–Ag shows that the mean diameter of Ag or Ag_2_O NPs is ∼10 nm.

## Conclusions

In this work, we have designed and synthesized copper-chlorophyllin-bonded carbon dots (CCPh-CDs) through a simple organic reaction at room temperature. The content of CCPh molecules on CDs has been tuned by modifying the concentration of CCPh in reaction. By comparing the PL and UV-Vis absorption spectra of CCPh-CDs with different CCPh contents, it is observed that CCPh significantly decreases the PL intensity of CDs, inferring that under ultraviolet irradiation, both electron transfer and FRET dual-behaviors occur from CDs to CCPh. Meanwhile, the measured luminescent decay spectra of CCPh and CCPh-CDs are 3.20 ns and 12.64 ns, respectively, indicating the electron transfer between the linked CCPh and C-dot. Based on the CCPh-CDs, Ag/Ag_2_O nanoparticles with the mean diameter of ∼10 nm can be obtained under ultraviolet irradiation, and further, they are measured by XRD, PL, FTIR and TEM. Such a combination of CCPh-CDs with Ag/Ag_2_O may significantly extend the application of C-dots with CCPh.

## Author contributions

The manuscript was written through contributions of all authors. All authors have given approval to the final version of the manuscript.

## Conflicts of interest

The authors declare no competing financial interest.

## Supplementary Material

RA-010-D0RA04958A-s001
