# Supplementary material for: Electron/energy co-transfer behavior and reducibility of Cu-chlorophyllin-bonded carbon-dots
Source: RSC Adv. 2020 Aug 26;10(52):31495–501. doi: 10.1039/d0ra04958a (PMC9056392; doi:10.1039/d0ra04958a)
Supplement: RA-010-D0RA04958A-s001 [file RA-010-D0RA04958A-s001.pdf]

Electronic Supplementary Material (ESI) for RSC Advances.
This journal is © The Royal Society of Chemistry 2020

## Supporting Information

# Electron/Energy Co-transfer Behaviors and Reducibility of Cu-chlorophyllin-bonded Carbon-dots

Tian-Hao Ji,*,† Xue-Li Li, † Yong-Yun Mao,‡ Zhi-Peng Mei, ‡ and Yan-Qing Tian*,‡

†Science College, Beijing Technology and Business University, Beijing 100048, China;

‡Department of Materials Science and Engineering, Southern University of Science and Technology, Shenzhen 518055, China

**Experimental section, PL spectra, and FTIR spectra**

**Experimental section**

**Preparation of CDs.**

At room temperature, citric acid (10.0 g) was first dissolved in DI-water (3.5 mL), and then ethylenediamine (3.0 mL) was added into the solution with stirring. The mixture solution was transferred to a poly(tetrafluoroethylene) (Teflon)-lined autoclave (30 mL) and heated at 180 ºC for 20 h. After the reaction, the reactor was cooled down to room temperature naturally. The obtained product was poured into anhydrous ethanol (300 mL) at room temperature with stirring, and after many hours, the mixture was centrifuged and washed by ethanol for at least four times. Finally, the prepared CDs were stored in ethanol before use.

**Preparation of CCPh-CD2 or CCPh-CD3**

Cu-chlorophyllin (135.0 mg or 70.0 mg), S-NHS (800.0 mg) and EDC (350.0 mg) were dissolved in 60 mL DMSO with stirring at room temperature, and then the CDs (500.0 mg) were added into the solution with stirring for 24 hours under light-proof condition. The obtained solution was sedimented using ethanol and centrifuged, and then washed by ethanol for three times. Finally, the solid product was vacuum-dried for 10 hours at 40 ℃, and denoted as CCPh-CD2 (0.70 wt.% of $Cu^{2+}$ content) or CCPh-CD3 (0.29 wt.% of $Cu^{2+}$ content).

**Measurements of PL spectra**

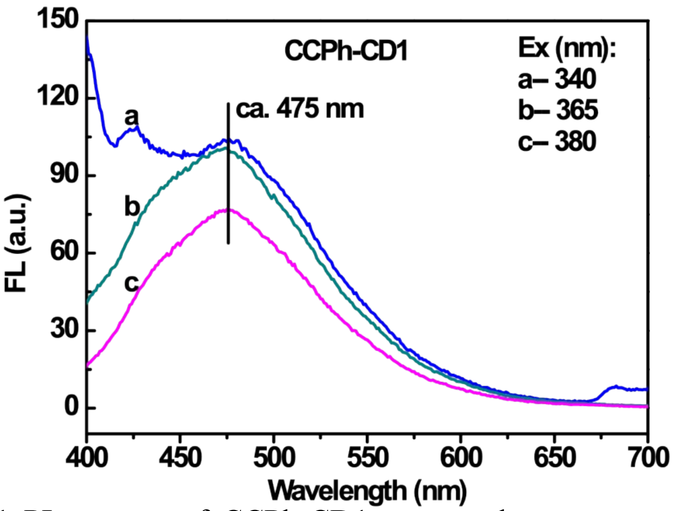


**S-Fig. 1** PL spectra of CCPh-CD1 measured at room temperature under different excitation wavelength.

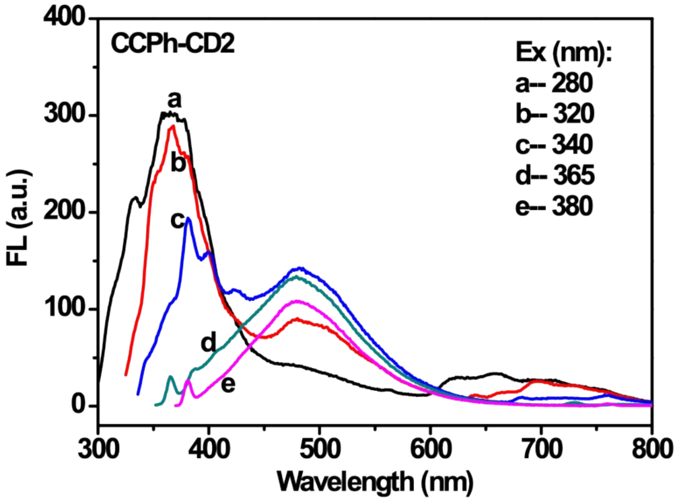


**S-Fig. 2** PL spectra of CCPh-CD2 measured at room temperature under different excitation wavelength.

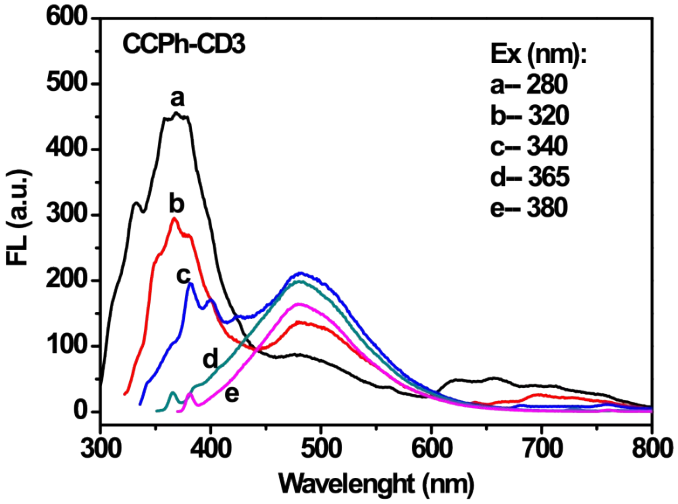


**S-Fig. 3** PL spectra of CCPh-CD3 measured at ambient temperature under different excitation wavelength.

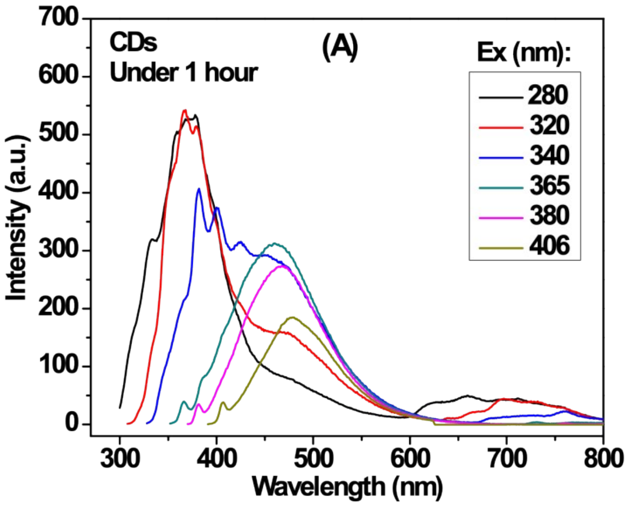


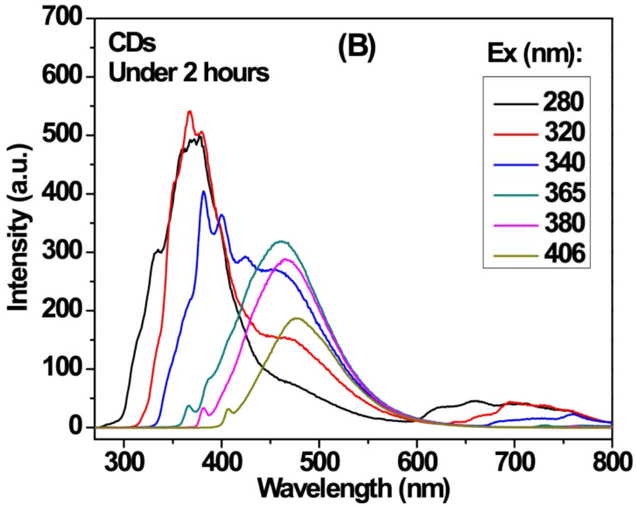


**S-Fig. 4** PL spectra of CDs under the ultraviolet light irradiation of 360 nm for (A) one hour and (B) two hours.

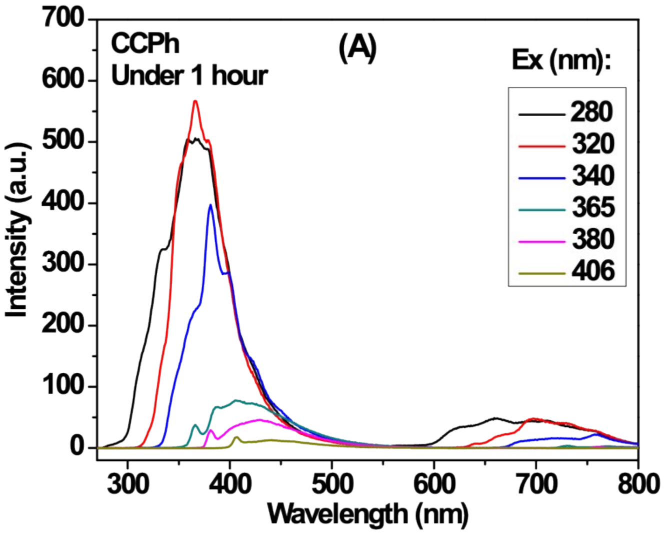

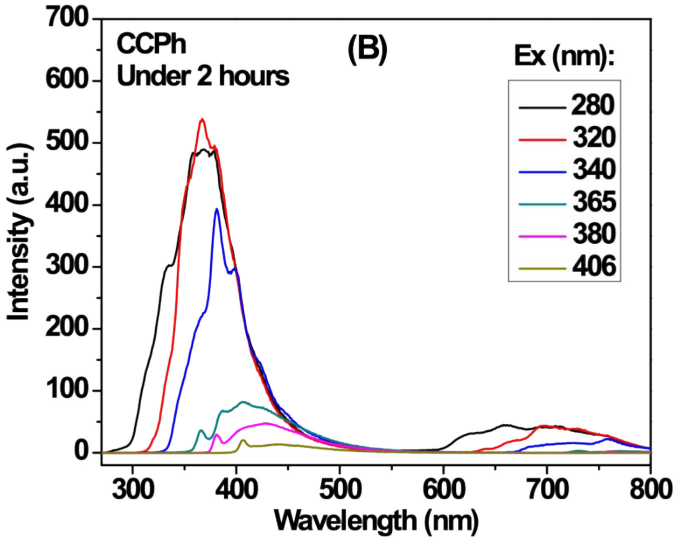


**S-Fig. 5** PL spectra of CCPh dissolved in DMF under the ultraviolet light irradiation of 360 nm for (A) one hour and (B) two hours.

## Measurements of FTIR spectra

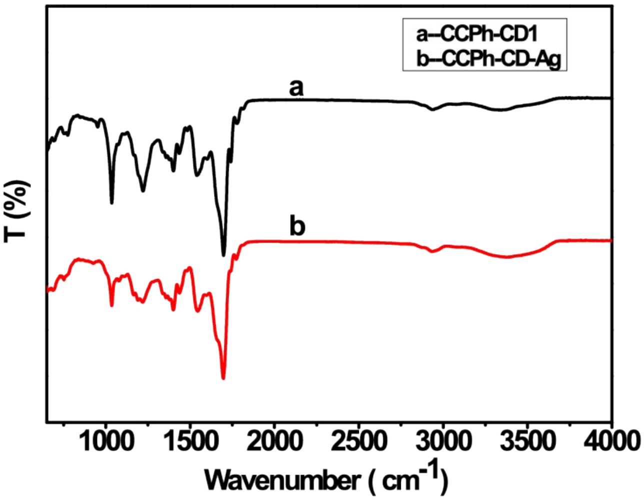


**S-Fig. 6** FTIR spectra of CCPh-CD1 and CCPh-CD-Ag measured at ambient temperature.
